# WHO WORKS LONGER IN LATER LIFE? KEY VARIATIONS IN WORKING LIFE EXPECTANCY IN INTERNATIONAL PERSPECTIVE

**DOI:** 10.1093/geroni/igad104.1045

**Published:** 2023-12-21

**Authors:** Brian Beach, Hugo Westerlund

## Abstract

In the context of population aging, policymakers are eager to encourage and enable longer working lives, assuming this will lead to increased tax revenue and reduced demand on social security and health care systems. Simultaneously, individuals who work longer will gain additional income and may experience the psychosocial benefits that come from workplace engagement. However, many factors drive early labor market exit, and blanket policy strategies to extend working lives often ignore the nuances that shape the length of real working lives. Such policies have emerged partly from increased life expectancies, although these measures fail to capture subgroup differences in the capacity to continue working. They also do not account for often discontinuous labor market trajectories, particularly around retirement. As such, scholars have turned to more refined methodologies and concepts like working life expectancy (WLE) to better inform policies that actively aim to increase retirement ages. This symposium highlights an ongoing program of work by the IDEAR network, which examines WLE using longitudinal studies of aging in Europe. The symposium features results from Sweden and England, estimating WLE using multi-state models. Unlike other approaches to estimate life expectancy employing the Sullivan method, this approach allows for reversible transitions between work and non-work, reflecting the dynamic and complex nature of modern labor market transitions and exits. The symposium further presents how WLE varies according to key determinants, including socioeconomic status, healthy lifestyles, and unpaid caregiving, highlighting the policy implications that emerge from recognizing inequalities and variations in WLE across vulnerable subgroups.

